# Training under planetary risk: planetary health ethics in clinical education and governance

**DOI:** 10.1186/s12909-026-09424-z

**Published:** 2026-05-21

**Authors:** Animesh Ghimire, Mamata Sharma Neupane

**Affiliations:** 1 Research Fellow, Sustainable Environment Foundation Nepal, Baneshwor-31, Kathmandu, Nepal; 2 School of Public Health, Chitwan Medical College, Bharatpur-5, Kailashnagar, Chitwan Nepal; 3 School of Nursing, Chitwan Medical College, Bharatpur-5, Kailashnagar, Chitwan Nepal

**Keywords:** Planetary health ethics, Medical education, Nursing education, Nepal, Climate change, Disaster preparedness, Health systems

## Abstract

**Background:**

Health-professional training in many low- and middle-income countries (LMICs) unfolds amid long-standing yet intensifying environmental, infrastructural, and health-system stressors, including extreme heat, unreliable power and water supplies, seasonal surges in vector-borne disease, and seismic risk. Yet undergraduate ethics teaching often remains encounter-centred, offering limited preparation for safe, fair, and role-bounded action when clinical systems are strained. This study examined how final-year medical and nursing students in Nepal understand these conditions and what they identify as practical entry points for planetary health ethics education.

**Methods:**

We conducted a qualitative descriptive study at a tertiary public institution in Madhesh Province, Nepal. Twenty-one final-year students participated in semi-structured interviews: 10 Bachelor of Science in Nursing students and 11 Bachelor of Medicine, Bachelor of Surgery students. Data were analysed using the Framework Method, combining inductive coding with sensitising concepts from planetary health ethics.

**Results:**

Three practice-proximal themes were identified. First, students described planetary disruptions as the “new clinical context”: clinical learning occurred amid overlapping pressures, including climate-exacerbated heat, dengue-season surges, intermittent power and water supply, cold-chain and equipment vulnerability, and earthquake-readiness routines. Their accounts emphasised supervised safety work: noticing risks early, adjusting tasks within scope, documenting clearly, and escalating through established channels. Second, professional futures were shaped by safety, reciprocity, and conditional return. Speciality and migration narratives were grounded less in abstract planetary ethics than in occupational safety, remuneration, family responsibility, reliable training, usable expertise, functioning equipment, and institutional readiness. Third, students identified a curricular gap and proposed concrete solutions, including locally grounded cases, hazard thresholds, escalation routes, Objective Structured Clinical Examination (OSCE) stations that simulate system failures, interprofessional drills, and the inclusion of situated expertise from staff who manage the material conditions of care.

**Conclusions:**

Planetary health ethics becomes educationally meaningful when translated into teachable and assessable routines for clinical practice under constraint. Programmes should prepare students to recognise hazard thresholds, communicate transparently, document constrained decisions, escalate appropriately, and learn from the infrastructures and personnel that make safe care possible. High-hazard LMIC settings are not peripheral to planetary health ethics; they offer vital insight into educating health professionals for just, resilient, and context-responsive care.

## Introduction

Health-professional education increasingly unfolds in clinical systems where environmental, infrastructural, and social stressors are not exceptional disruptions but recurrent conditions of care. The convergence of climate change, biodiversity loss, and pollution—the so-called “triple planetary crisis”—is reconfiguring the conditions under which health is produced, protected, and lost [1]. The interplay of heat extremes, hydrometeorological variability, and compromised infrastructure significantly influences the clinical case mix, continuity of healthcare services, and occupational safety. These factors also exacerbate prolonged risks to mental health and the trajectories of non-communicable diseases [2–4]. However, such pressures should not be attributed solely to climate change. In Nepal and other hazard-exposed low- and middle-income countries (LMICs), health-service instability also reflects long-standing seismic exposure, infrastructural fragility, energy interruptions, supply-chain dependence, governance constraints, labour mobility, and social inequities; climate change is therefore best understood as an emerging and intensifying force that compounds some of these vulnerabilities—particularly heat, hydrometeorological stress, air-quality deterioration, and vector-borne disease—rather than as their singular cause [5]. Health systems play a significant role in perpetuating environmental challenges, as their operational footprint often contradicts their fundamental mission of promoting health and well-being. Moreover, these systems exhibit considerable variability in their preparedness to effectively mitigate and adapt to emerging environmental hazards [6]. This is not merely a technical or an operational challenge but an ethical one: decisions about who is protected first, how harms are distributed across patients and time, and what forms of advocacy are legitimate within institutions have become routine features of everyday care. A contemporary bioethics adequate to this reality must therefore look outward—from encounter-level dilemmas to the infrastructures, ecologies, and governance arrangements that condition them [7, 8].

Historically, bioethics emerged at the intersection of medicine, research, and law, crystallising around the principles of autonomy, justice, beneficence, and non-maleficence in clinical and experimental settings [9, 10]. Over subsequent decades, *public health ethics* developed as a cognate field concerned with population-level risks, social justice, and solidarity [11], while environmental ethics foregrounded obligations to non-human life and ecosystems [12]. Although the earliest visions of bioethics were expansive—connecting human flourishing to the biosphere [13]—these strands largely diverged, resulting in contemporary biomedical ethics concentrating on individual rights, while *environmental ethics* focuses on the intrinsic value of nature [14]. Recent scholarship urges a reconnection. Public health ethics is well placed to bridge the domains by linking the health of individuals to that of populations, animals, and environments, and by re-centering questions of equity and collective responsibility [8]. At the same time, critical voices have argued that bioethics risks stagnation when it collapses into medical ethics and remains captive to unexamined dogmas [13]; renewal requires turning towards the methods and concerns of public health ethics and engaging explicitly with contemporary planetary conditions [7]. For undergraduate medicine and nursing, the practical ethical deficit is therefore precise: students are commonly taught to reason through consent, confidentiality, autonomy, and bedside allocation, but receive far less structured preparation for ethically bounded action when heat, water reliability, power supply, infection surges, equipment failure, or evacuation risk alter what safe and fair care requires.

Within this reconnection agenda, *planetary health ethics* has been advanced as a necessary enlargement of bioethics: an approach that integrates human welfare with biosphere health, centres intergenerational and distributive justice, takes multispecies and biodiversity considerations seriously, embraces precaution under uncertainty, and legitimises professional advocacy within institutions and governance. This lens also insists on epistemic inclusion—amplifying youth, Indigenous, and other marginalised voices—and on aligning pedagogy, regulation, and operational practice so that ethical commitments are actionable rather than aspirational [15]. Yet the ethical landscape around climate and health remains fragmented and under-developed: action-guiding resources for practitioners are scarce; ethical analysis is thin across core environmental domains (energy, food, water, transport, pollution); research on climate responses seldom includes ethical reflection; and low-income countries are under-represented in both empirical study and normative debate. A synthesis that can guide decisions where human and non-human interests collide and where the claims of future generations weigh on present practice remains emergent [7]. In this study, planetary health ethics is not used as a broad normative overlay or as a substitute for disaster ethics or public health ethics. Disaster ethics is indispensable for acute crisis standards and emergency preparedness, and public health ethics remains central to population risk, solidarity, and justice; planetary health ethics is used here because it connects these concerns to ecological interdependence, healthcare’s own environmental footprint, intergenerational responsibility, and the ethical significance of learning from communities already practising under chronic multi-hazard instability [16, 17]. Operationally, it functions as a sensitising framework that directs analytical attention to how medicine and nursing students navigate care provision amidst systemic constraints. It provides a lens to systematically examine both their frontline decision-making processes and the educational architectures that shape those competencies.

**Fig. 1:**
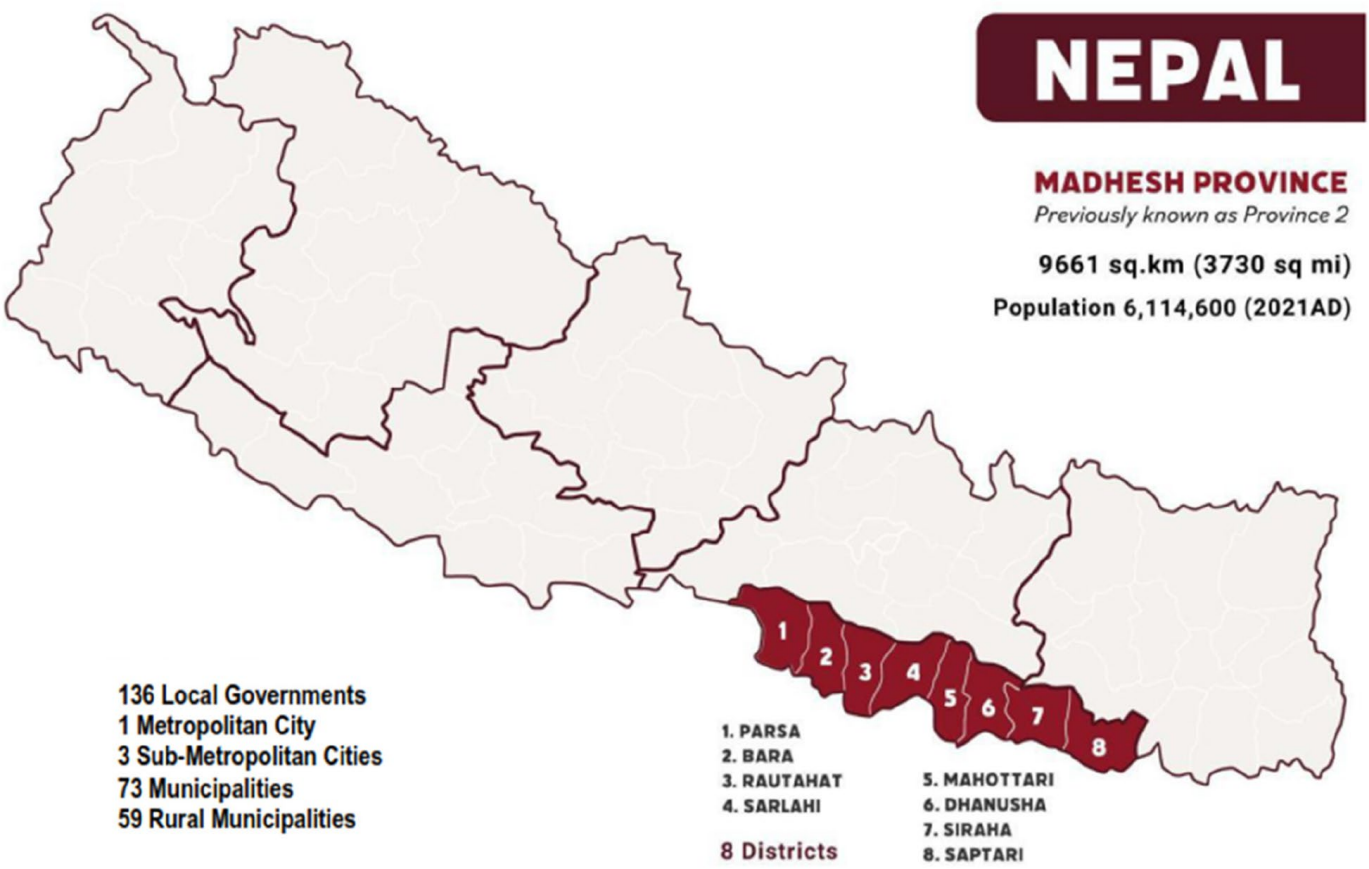
Madhesh province map and data [21]

Nepal offers a salient context for this inquiry. Home to approximately 29.6 million people and spanning an extraordinary altitudinal gradient from subtropical lowlands (< 100 m) to the Himalayan crest (8,849 m) [18, 19]. The country exhibits strong climatic contrasts yet has warmed across all zones, with mean annual temperatures increasing by around 0.06 °C per year since the late 1970 s, and high-mountain regions already exceeding the global warming average [20]. The southern lowlands, known as the Terai region, comprise roughly a quarter of the national land area but over half the population; within the eastern Terai, Madhesh Province forms a 400-km corridor along the Indian border [21] (Fig. 1). Although it accounts for 6.5% of the national territory, Madhesh is the most densely populated province, experiences routine summer temperatures above 40 °C, and faces recurrent monsoon flooding [21]. Moreover, vector-borne diseases—including dengue—have become a major public health concern, with large outbreaks during 2022–2024 disproportionately affecting southern Nepal, particularly Madhesh province [22, 23]. Alongside thermal and hydrological hazards, Nepal is a seismic hotspot. After a devastating earthquake measuring 7.8 that struck Nepal in 2015 [24], the recent 2023 earthquake of 6.4 magnitude has exposed the compounding health effects of seismic shocks—access disruptions, infectious-disease risk, mental-health issues, and supply-chain fragility—and the need for preparedness that expands from building codes and logistics to risk communication and community engagement [25]. Madhesh is therefore not presented here as a setting where climate change alone newly produces clinical adversity. Rather, it is a high-hazard and structurally constrained clinical education environment in which climate-exacerbated heat, flooding, and vector dynamics intersect with seismic risk, infrastructure limitations, resource scarcity, and health-system fragility. This makes it an empirically important site for examining how near-graduation students learn to interpret, prioritise, document, and escalate risk when safe care depends as much on systems and surroundings as on individual clinical judgement.

Despite this reality, ethics education in undergraduate medicine and nursing globally—especially in LMIC settings—often remains anchored in classical, encounter-centred bioethics, with limited attention to hazards, infrastructures, or the distribution of risk across patients, time, and communities [26–29]. The literature repeatedly documents four gaps. First, *conceptual*: there is a shortage of integrated, action-guiding frameworks that link clinical decisions to planetary considerations in ways trainees can use. Second, *empirical*: LMIC-situated qualitative evidence on how students actually experience hazard-conditioned practice, and how they construe legitimate action within scope, is scarce. Third, *pedagogical*: where planetary content appears, it is frequently elective, de-contextualised, or siloed from assessment, leaving the hidden curriculum to define advocacy boundaries and fairness criteria. A fourth *transition‑to‑practice gap* concerns how near‑graduation learners perceive the lack of planetary health content in their curricula and how they expect this to influence their readiness for practice—an area that remains largely undocumented. Recent reviews reinforce these gaps: climate and planetary health competency frameworks remain inconsistently aligned and are particularly limited in relation to the Global South, while interprofessional climate-health curricular interventions remain few and unevenly evaluated [30, 31]. Evidence from health professions education in other LMIC or middle-income contexts also shows that planetary health and sustainable healthcare content are emerging but remain unevenly distributed, variably assessed, and often dependent on local institutional initiative rather than on embedded curricular design [32]. This fragmentation is especially problematic in regions where heat, power outages, vector surges, and seismic risk are the clinical baseline, not the exception.

Addressing these gaps requires more than importing planetary health ethics into LMIC curricula as an external framework. High-hazard LMIC settings offer an epistemically important vantage point for planetary health ethics because trainees and clinicians in such settings have long confronted ethical problems that climate change is making more visible across health systems globally: sustaining care in unstable facilities, allocating scarce resources fairly, protecting vulnerable patients during environmental stress, and negotiating professional futures under structural constraint. Accordingly, this study focuses empirically on final-year medical and nursing students’ accounts of clinical learning in one high-hazard institution; it does not directly audit curricula, evaluate educators, assess formal assessment systems, or examine governance processes. Broader implications for curriculum reform and institutional preparedness are therefore developed from students’ practice-proximal accounts rather than treated as direct institutional findings. This study is guided by two research questions (RQ) and one objective:

- RQ1: How do final-year medical and nursing students in a high-hazard LMIC setting understand and navigate ethical challenges that arise when environmental, infrastructural, and resource constraints affect clinical learning and patient care?
- RQ2: What curriculum and assessment strategies do students identify as useful for preparing graduates for safe, fair, and role-bounded action under such conditions?
- Objective: To generate student-informed, practice-proximal recommendations for integrating planetary health ethics into undergraduate medical and nursing education in ways that are contextually grounded, clinically usable, and proportionate to the evidence generated by this research.

## Methods

### Study design and theoretical framework

Grounded in an interpretive paradigm, which seeks to understand the meanings individuals construct within their socio-cultural and institutional contexts, this study adopted a qualitative descriptive design to elicit practice-proximal accounts from final-year undergraduate nursing (UNS) and medicine (UMS) students in a multi-hazard setting [33]. A qualitative descriptive design was selected to keep the analysis close to participants’ language while permitting a modest interpretive lift when patterns bear directly on curriculum, assessment, and governance in clinical education [34]. This design was therefore appropriate for examining how students described, interpreted, and responded to clinical learning under recurrent environmental, infrastructural, and resource constraints, without directly evaluating institutional curricula, educator practices, assessment systems, or governance processes. Consistent with qualitative description [33], the purpose was to produce a coherent, practice-near account of participants’ experiences in terms that remain recognisable to the clinical and educational settings from which they were generated.

The study was theoretically anchored in *planetary health ethics* as articulated by Anderson et al. [15] *Bioethics for the Planet*, which broadens conventional bioethics by integrating human health with biosphere health, centring distributive and intergenerational justice, recognising more-than-human and biodiversity concerns, adopting precaution in conditions of uncertainty, legitimising advocacy within professional roles, and embedding ethics in training and governance, with explicit inclusion of youth and other marginalised voices. Because the empirical scope of this study was students’ clinical learning rather than programme-level governance, we used selected components of planetary health ethics that were most relevant to practice-proximal education: hazard-aware reasoning, fairness under constraint, role-bounded escalation and advocacy, environmental externalities visible in care, and curricular strategies that could make these capacities teachable and assessable. In practical terms, this lens functioned as a set of sensitising concepts for the study—informing the construction of the semi-structured interview guide, shaping criterion-based purposive sampling of final-year trainees with diverse hazard exposures, and guiding the initial deductive nodes in the *framework analysis* [35, 36]—while leaving ample space for inductive coding and theory–data dialogue throughout [37]. The framework, therefore, oriented attention but did not determine the findings: students’ descriptions of clinical practice, safety work, resource constraints, supervisory boundaries, and perceived curricular gaps were first analysed on their own terms before being interpreted cautiously through planetary health ethics. Broader implications for public health ethics, regulatory reform, institutional governance, and healthcare’s environmental footprint are treated as interpretive implications rather than as direct empirical claims from this single-site student interview dataset.

### Sample, setting, and participant recruitment

The study was conducted at a tertiary public institution with an affiliated teaching hospital in Madhesh Province, located in south-eastern Nepal [38]. This semi-urban area routinely experiences summer temperatures exceeding 40 °C, monsoon-related flooding, recurrent surges of vector-borne diseases, and significant seismic risk [21]. The setting was conceptualised as a structurally constrained, multi-hazard clinical education environment in which climate-exacerbated heat, flooding, and vector-borne disease intersect with non-climatic hazards and health-system vulnerabilities, including seismic risk, power interruptions, water unreliability, maintenance constraints, and resource scarcity. This framing was used to avoid attributing students’ experiences solely or primarily to climate change and to situate the data within a broader ecology of chronic environmental and infrastructural instability. Consequently, the institution was purposefully selected because it trains large cohorts in Bachelor of Science in Nursing (BSc Nursing) and Bachelor of Medicine, Bachelor of Surgery (MBBS), ensuring access to final‑year students with substantial clinical exposure across wards and various specialities.

We used criterion‑based purposive sampling to identify eligible participants who could speak to the research aims [39]. Inclusion criteria were: (1) enrolment in the final year (UNS Year 4; UMS Year 5); (2) ≥ 18 years of age; and (3) willingness to discuss experiences and views related to planetary health ethics as encountered in training and placements. Final-year students were selected because they had accumulated substantial ward-based exposure, had experience of supervised participation in clinical routines, and were approaching transition to internship, employment, or postgraduate training; their accounts were therefore well suited to exploring learner perspectives on preparedness, scope, escalation, and perceived curricular relevance. However, this sample was not designed to represent institutional leadership, educators, registered clinicians, curriculum committees, or regulatory bodies, and the findings should not be read as a direct audit of formal curriculum or governance arrangements. To optimise heterogeneity of experience, we sought maximum variation [40] across gender, urban/rural family background, and recent exposure to salient hazards (e.g., heatwaves, dengue‑season surges, outages, the 2015/2023 earthquake).

Recruitment proceeded in three linked stages that combined purposive and convenience strategies with participant‑led referral [39, 41]. First, an announcement flyer (with a secure QR code link) describing the study aims, inclusion criteria, confidentiality, and voluntariness was posted in student‑facing locations (library, common rooms, departmental noticeboards). Students who self‑registered via the QR code constituted a convenience pool that met the criterion frame; the Principal Investigator (AG) verified eligibility and arranged interviews. Second, to maximise variation, we monitored emerging sample characteristics and invited additional volunteers who met underrepresented criteria. Third, after interviews, we used participant‑led referral, referred to as snowball sampling [42] to identify peers who met the inclusion criteria and were likely to offer distinct perspectives (e.g., different clinical rotations or hazard encounters). The final sample comprised 21 students—10 UNS and 11 UMS—reflecting the gender balance and programme distribution described in the Results section.

### Data collection

Data were collected from April to June 2025 through face-to-face, in-depth, semi-structured individual interviews lasting 45 min, which were conducted by the first author (AG), a clinician–academic experienced in qualitative inquiry. To minimise power differentials, the interviewer had no instructional, supervisory, or assessment role with participants. Interviews took place in private rooms on campus at times convenient to participants. Before each interview, written informed consent was obtained using a standard script that covered the study purpose, voluntariness, confidentiality, the right to withdraw, and audio recording.

Interviews were conducted in Nepali and/or English based on participant preference. It is important to note that English is a primary medium of instruction in Nepali higher education, including medicine and nursing. The semi‑structured guide (Table 1) was developed from the research aims and a targeted review of the literature in planetary health ethics, public health ethics, disaster and climate impacts on health services, and health‑professional education. The guide was designed to elicit students’ accounts of clinical learning and perceived educational needs, not to examine official curricula, educator intentions, assessment blueprints, hospital policy, or governance processes.

**Table 1:** Semi-structured interview guide outline

Questions	Interview question (prompts in italics indicate optional probes or brief vignettes)
1	Tell me about a recent clinical day that was affected by heat, power or water interruptions, or dengue-season surge. *Which tasks changed first? Who directed those changes?*
2	When ward temperatures rose above usual levels or air-conditioning was ineffective, what adjustments did you observe or assist with? *Scheduling, medication timing, vital-sign frequency?*
3	*Vignette: 41 °C heat with a 90-min outage.* Under supervision, what are the first safety checks you would help with? *Patients/devices to prioritise; documentation; escalation route*
4	Describe any earthquake-readiness drills or actions you practised after the 2023 event. *Roles, evacuation paths, and communication with families*
5	Think of a time when resources felt tight (e.g., PPE shortages, generator capacity). What fairness judgments did the team make, and what did you learn about your role?
6	When environmental or infrastructure issues posed risks, how did you raise concerns and to whom? *What counted as legitimate advocacy for a student?*
7	How, if at all, has ethics teaching prepared you for decisions under heat, outages, water reliability, and vector surges? *Examples of topics missing or most useful*
8	What curricular changes would make you more confident in these situations?
9	Looking ahead, how do system conditions (safety, reliability, maintenance) influence your speciality and migration plans? *Conditions that would support return or long-term stay*
10	In your view, what should be the graduate competencies for planetary health ethics in this setting?

Questions explored: (a) clinical learning under heat, outages, water reliability, vector surges, and seismic events; (b) perceptions of responsibility and fairness when resources are constrained; (c) boundaries of role‑appropriate advocacy and escalation pathways; and (d) curricular experiences and suggestions for teach‑and‑assess entry points. Consistent with the empirical scope, prompts were phrased to foreground supervised, role-bounded student action—what participants observed, assisted with, documented, escalated, or believed should be taught—rather than assuming that students had authority over institutional decisions.

We also used brief local vignettes (e.g., a heatwave with rolling power cuts, a cold‑chain alarm during an outage, personal protective equipment (PPE) shortages, smoke drift from waste handling, earthquake‑prompted drills) to elicit reasoning within students’ scope of practice. These vignettes deliberately included both climate-exacerbated hazards and non-climatic infrastructural or seismic disruptions, enabling participants to discuss multi-factorial instability without requiring them to attribute clinical challenges to climate change alone. The guide was pilot‑tested with two students (not included in the final sample) for clarity and sequencing; minor wording adjustments were made.

Audio recordings were transcribed verbatim by AG. Segments in Nepali were transcribed in Nepali and then translated into English by AG; the second author, MSN, bilingual in Nepali/English, cross‑checked a subset for fidelity and conceptual equivalence. All transcripts were de‑identified and stored on a secure, access‑restricted institutional server. As part of member checking, participants were offered concise summaries of their interviews to confirm accuracy and resonance; clarifications were incorporated where relevant [43].

### Researcher characteristics and steps to minimise influence

The research team comprises clinician–academics with backgrounds in nursing, medicine, public health, and prior work on disaster education and ethics in LMIC settings. We documented assumptions and decision points (e.g., anticipated curricular gaps, expectations about hazard-aware practice and escalation) in working notes and discussed these during analysis to consider how positioning might shape coding or emphasis. This reflexive process was particularly important because the study used planetary health ethics as a sensitising framework; the team therefore scrutinised whether theoretical commitments were illuminating participants’ accounts or overextending beyond them. The interviewer’s non-teaching status, standardised consent script, private interview spaces, and the use of a semi-structured guide aimed to reduce social desirability pressures and maintain consistency across interviews. Reflexivity was treated as an ongoing analytic practice rather than a one-off declaration of positionality [44].

### Data analysis

We applied the Framework Method because of its clear, auditable stages and its fit for studies with applied objectives in healthcare education [36]. The method was also appropriate because it permits systematic comparison across cases while accommodating both deductive and inductive forms of analysis, a requirement for this study’s theory-informed but data-led design [45]. Analysis began with familiarisation, during which both authors read all transcripts closely and noted preliminary ideas around hazard signals, supervisory instructions, fairness judgements, advocacy channels, and curriculum touchpoints. Guided by the research aims and familiarisation notes, we developed an analytical framework (coding index) that combined deductive domains drawn from planetary health ethics (e.g., precaution under uncertainty, distributive and intergenerational justice, environmental externalities and multispecies considerations, advocacy within governance) with inductive codes grounded in the Nepali training context (e.g., heat–outage workflow, PPE stewardship during shortages, cold‑chain vigilance, triage management, drills and escalation roles).

The analysis was organised in three explicit tiers. The first tier was descriptive, preserving what participants reported observing, doing, or being instructed to do. The second tier examined participant meaning, including how students understood safety, fairness, responsibility, professional boundaries, and preparedness. The third tier involved cautious authorial interpretation, in which selected patterns were considered in relation to planetary health ethics only when the underlying descriptive and meaning-oriented evidence supported that interpretive move. Thus, pragmatic clinical accounts—such as rescheduling wound care during heat, switching to gravity infusions during outages, documenting cold-chain alarms, or escalating crowding concerns—were not automatically coded as planetary health ethics. They were first retained as practice-proximal safety work and only later considered ethically salient where they raised questions of fair allocation, precaution, scope, or accountability. Similarly, references to migration, speciality choice, or institutional reliability were initially coded as occupational safety, professional development, family obligation, or health-system constraint, rather than presumed to represent planetary ethics.

To establish a shared application of the framework, both authors independently indexed an initial subset of transcripts, then met to compare coding decisions, resolve discrepancies through discussion, and refine code definitions and inclusion/exclusion criteria. In these meetings, the deductive categories of planetary health ethics were approached as analytical questions rather than definitive codes. For instance, the team explored whether the quotes pertained to themes such as fairness under constraint, environmental externality, role-bounded advocacy, or future-oriented responsibility. However, participants' narratives were also allowed to reside within descriptive or contextual categories when ethical interpretation was not warranted. The agreed codebook was then applied to the remaining transcripts, with periodic calibration checks to sustain consistency. All coding and charting were conducted manually; no qualitative data analysis software was used. We next undertook charting in a case‑by‑code matrix, entering synthesised summaries and short verbatim anchors for each participant under each relevant code. This matrix enabled systematic comparison within and across UNS and UMS accounts and made the convergent, variant, and negative cases visible. Negative and disconfirming cases were used to test whether the emerging themes overstated the role of climate change, planetary health ethics, or student agency; where participants foregrounded remuneration, workplace safety, family obligations, supervision, or infrastructure rather than planetary reasoning, those meanings were retained rather than subsumed under the theoretical framework.

The final stage involved mapping and interpretation: reviewing matrix patterns, examining intersections among hazard signals, supervisory instructions, fairness logics, and advocacy pathways, and testing candidate configurations against the full dataset. Through iterative discussion, we arrived at a thematic structure that captured patterned regularities while preserving discipline‑specific nuances and scope‑of‑practice boundaries. The development of themes, therefore, evolved from empirical regularities observed in students' accounts towards a careful conceptual interpretation, rather than relying on predefined categories of planetary health ethics. Table 2 clarifies this analytical process by illustrating the progression from meaning units to codes, subcategories, categories, and themes. We judged analytical sufficiency pragmatically, consistent with the Framework Method’s applied orientation: once the coding framework and matrix adequately captured the conceptual range pertinent to the research questions and additional indexing yielded minimal modification, we proceeded to stabilise the thematic account. We distinguished this judgement from claims of universal saturation or theoretical exhaustiveness. Instead, we assessed whether the dataset provided sufficient information power and analytic depth to answer the narrowed research questions about student experiences, perceived preparedness, and curriculum-relevant suggestions within this setting [46, 47].

**Table 2:** Data analysis process

Meaning Unit	Code	Sub-Categories	Categories	Theme
“On hot days we were instructed to bring wound care forward to the morning and […] preceptor and reassessed observations more frequently” (UNS 04, Y4)	Heat-aware workflow adjustment	Morning rescheduling; Observation frequency; Preceptor-led escalation	Hazard-aware clinical routines	Theme 1 — Planetary disruptions as the “new clinical context”
“Under the SRMO’s supervision, we prioritised initial vitals and hydration, […] triage is different from the first minute avoid overcrowding […]” (UMS 15, Y5)	Triage recalibration under heat stress	Prioritise high-risk; Early escalation; Discharge pacing to prevent crowding	Clinical risk stratification under environmental stress	Theme 1 — Planetary disruptions as the “new clinical context”
“When there is a power outage, nurses switch from the electric intravenous pump (IV) to the gravity line so that the generator sustains […].” — (UNS 09, Year 4)	Power-outage adaptation and equipment prioritisation	Shift to gravity infusion; Generator load protection; Maintain vital devices	Infrastructure-conscious care	Theme 1 — Planetary disruptions as the “new clinical context”
“I want to specialise abroad and then return. As a doctor here, you carry status but also obligation. In our hospitals, there is a lack of planet awareness […]” (UMS 19, Y5)	Conditional return framed as obligation	Skill acquisition abroad; Obligation to community; Planet-aware practice gap	Ethical calculus of mobility	Theme 2 — Professional futures amid planetary risk
“My plan is permanent migration. On the wards during dengue season with outages, staff safety felt uncertain—[…] thinking about coming back to work long term” (UNS 02, Y4)	Occupational safety as driver of permanence	PPE sufficiency; Rest and staffing; Supply reliability	Right to safe work	Theme 2 — Professional futures amid planetary risk
“What's the point of learning an advanced surgical technique abroad if I come back and the one machine we need is broken? We see it all the time […] somewhere else. That’s not just burnout; it's a moral injury.” — (UMS 13, Y5)	Reciprocity conditions for return; moral injury risk	Maintenance & parts; Usable roles; Protected teaching time	Justice-centred international partnership	Theme 2 — Professional futures amid planetary risk
"We learn about medical ethics from American and British textbooks. […] Our problems are different, but our ethics teaching is from another world." — (UMS 18, Y5)	De-contextualised ethics curriculum	Western cases dominate; Missing hazard contexts; Relevance gap	Curricular lacuna	Theme 3 — Navigating curricular gap with student-led solutions
“If there was an OSCE station where the system fails—heat, outage, short PPE—and we had to justify safe adjustments and choose the right escalation channel, we would prepare for it seriously” (UMS 14, Y5)	Assessment-driven competency building	System-failure scenarios; Justification of trade-offs; Correct escalation	Teach-and-assess entry points	Theme 3 — Navigating curricular gap with student-led solutions
“The most eye-opening session we had was when they invited cleaners, ward assistants, maintenance team, bio-medical engineers and hospital scientists to speak to us. […] who are on the frontline of those risks every single day.” (UNS 07, Y4)	Epistemic inclusion of situated expertise	Environmental services knowledge; Risk visibility; Respect for frontline roles	Ecology of care in education	Theme 3 — Navigating curricular gap with student-led solutions

### Rigour and trustworthiness

We addressed credibility, dependability, confirmability, and transferability to improve the rigour of this study [48]. Credibility was enhanced by member checking of interview summaries, peer debriefing with an independent scholar in planetary‑health education who reviewed methodology and emergent themes, and attention to negative cases during mapping. The use of negative cases was especially important for preventing overextension of planetary health ethics beyond the data and for preserving accounts in which students emphasised occupational safety, supervision, resource scarcity, remuneration, family obligation, or institutional fragility. Dependability was supported by a documented audit trail (sampling log; recruitment records; codebook versions; matrix iterations; analytic memos). Confirmability derived from transparent procedures—verbatim transcription, bilingual cross‑checks for translation, de‑identification, consensus‑based coding decisions, and secure data storage. Transferability is facilitated through a thick description of the setting, participant characteristics, hazard context, analytic procedures, and a full socio-demographic characteristic of the participants. Collectively, these procedures were designed to make visible how the study moved from student accounts to descriptive findings and then to bounded ethical interpretation.

### Ethical consideration

This study was conducted in accordance with the ethical principles outlined in the Declaration of Helsinki. Ethical clearance was obtained from the Nepal Health Research Council (approval number—133/2025).

## Results

### Participant characteristics

We interviewed 21 final-year undergraduates at a tertiary public institution in Madhesh province, Nepal: 10 Undergraduate Nursing students (UNS) studying BSc Nursing, a four-year degree (all women; Year 4), and 11 Undergraduate Medical students (UMS) pursuing a Bachelor of Medicine and Bachelor of Surgery (MBBS) degree (8 men, 3 women; Year 5), a five-and-a-half-year qualification. All participants were unmarried, had completed high school as their highest qualification on entry, and reported prior disaster exposure—most commonly heatwaves and the 2023 earthquake, as well as monsoon-related flooding and landslides. A clear majority had completed at least one rural clinical placement, and nearly all had experienced power outages during their placements; dengue season rotations and water insecurity were also common. Speciality aspirations were primarily focused on acute care (ED/ICU/OT/Anaesthesia), with a significant minority interested in internal medicine/paediatrics or surgical streams; a few prioritised community or primary care. Short-term migration intentions (up to 3 years) leaned towards seeking work or training abroad. Over a longer horizon, intentions varied by discipline: UMS students frequently expressed a desire to return to Nepal, citing prestige and service ideals, whereas BSc Nursing students more often anticipated permanent migration. The sample's exclusively female composition for UNS reflects the prevailing gender dynamics in Nepal's nursing landscape, where nursing is predominantly perceived as a female-dominated field. This observation is further supported by WHO observatory data, which indicate that the nursing workforce in Nepal is entirely female (WHO, 2023c), whereas the female medical workforce is 33% [49]. Hence, the participants' gender profile reflects the broader dynamics of the Nepali workforce. Interestingly, all 21 participants identified as Generation Z (Gen Z), referring to individuals born after 1995, often also called "digital natives" or "zoomers" [50]. Table 3 summarises variables relevant to our analytical aims.

**Table 3:** Socio-demographic characteristics of participants (*n* = 21)

**Participants Characteristics**	**Category or range**	***N* = 21 (%)**
Age (years)	18–24	18 (85.7)
	25–28	3 (14.3)
Gender identity	Woman	13 (61.9)
	Man	8 (38.1)
Programme & year	BSc Nursing, Year 4 (Y4)	10 (47.6)
	MBBS, Year 5 (Y5)	11 (52.4)
Locale (family home)	Urban	12 (57.1)
	Rural	9 (42.9)
First-generation college student	Yes	12 (57.1)
	No	9 (42.9)
Prior disaster exposure (any)	Yes	21 (100)
Salient recent hazard (self-reported; multiple responses)	2015/2023 earthquake	21 (100)
	Monsoon flooding/landslides	21 (100)
	Heatwave/air-quality events	21 (100)
Rural clinical placement exposure	Yes	21 (100)
Power outage during placement	Yes	21 (100)
Dengue-season placement	Yes	21 (100)
Water insecurity during placement	Yes	21 (100)
Speciality aspiration cluster	Acute/critical (ED/ICU/OT/Anaesthesia)	11 (52.4)
	Surgery/procedural	4 (19.0)
	Internal medicine/paediatrics	4 (19.0)
	Community/primary care/public health	2 (9.5)
Migration intention (≤ 3 years)	Seek work/training abroad	14 (66.7)
	Remain in Nepal	3 (14.3)
	Undecided	4 (19.0)
Long-term return intention (about 5-year horizon)	Yes	9 (42.9)
	No	7 (33.3)
	Undecided	5 (23.8)

## Findings

**Fig. 2:**
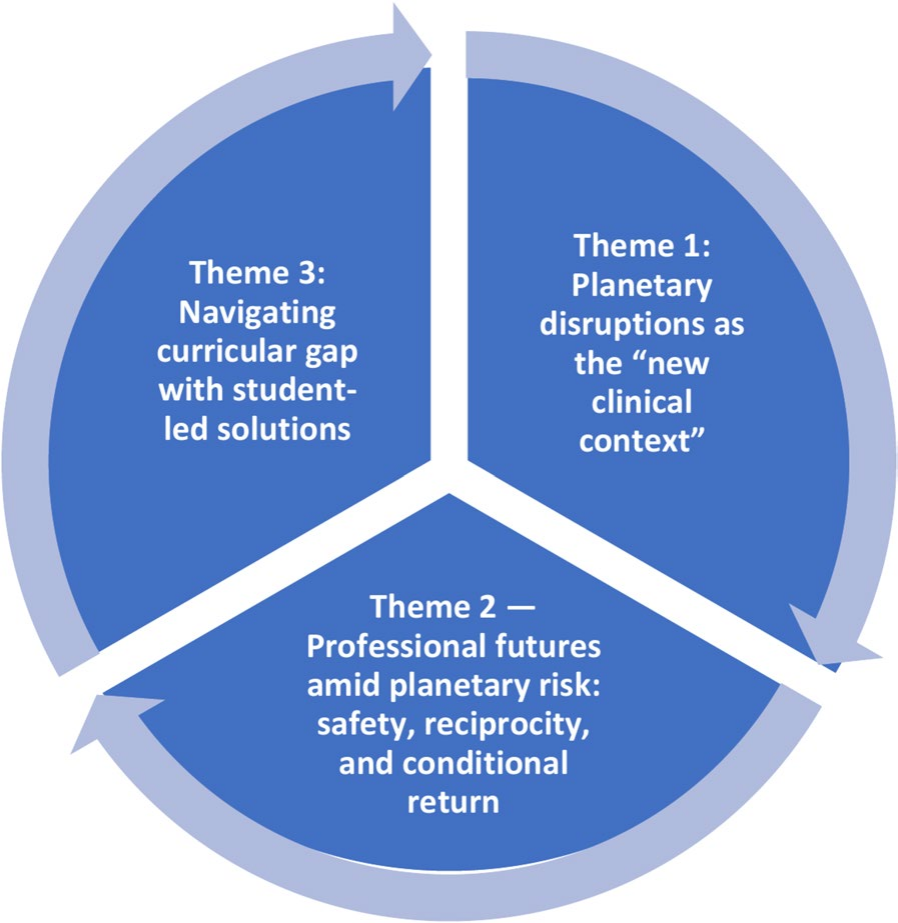
Theme map of findings: descriptive relationships among clinical learning under recurrent multi-hazard and infrastructural stressors (Theme 1), student accounts of professional futures shaped by safety, training quality, family obligations, usable expertise, and institutional readiness (Theme 2), and student-identified curriculum and assessment entry points for practice under constraint (Theme 3)

Three themes summarise how final-year nursing and medical students described learning, career planning, and curricular expectations within a clinical environment marked by recurrent environmental, infrastructural, seasonal, and seismic stressors (Fig. 2). Theme 1—Planetary disruptions as the “new clinical context”—presents students’ accounts of supervised safety work under overlapping pressures, including climate-exacerbated heat and dengue-season surges, intermittent power and water supply, equipment vulnerability, and earthquake-readiness routines, without treating these conditions as arising from a single cause. Theme 2—Professional futures amid planetary risk: safety, reciprocity, and conditional return—examines how these conditions entered students’ speciality and migration narratives, which participants grounded primarily in occupational safety, reliable training, remuneration, family responsibility, usable skills, institutional readiness, and conditions for return or permanence. Theme 3—Navigating curricular gaps with student-led solutions—reports students’ perceived gap between classical ethics teaching and the practical demands of ward-based learning under constraints, alongside their suggestions for locally grounded cases, hazard thresholds, OSCE stations, interprofessional drills, escalation pathways, and the inclusion of situated expertise. Taken together, the interrelated themes create a cyclical framework: engagement in practice amid planetary risk informs individuals' professional choices; these choices, in turn, signal the requisite competencies that training programs must deliver; consequently, structurally aligning education with these frontline realities equips the future workforce to sustain care within environmentally precarious health systems.

### Theme 1 — planetary disruptions as the “new clinical context”

In Madhesh Province, students described clinical training as taking place amid recurrent, overlapping stressors: climate-exacerbated heat, dengue-season surges, intermittent power and water supply, and seismic preparedness following the 2023 earthquake. Participants did not present these conditions as arising from a single cause. Rather, they described a clinical environment in which environmental pressures, infrastructure limitations, seasonal disease patterns, and disaster-readiness routines intersected in everyday ward work. What counted as a “normal” shift had already been recalibrated: students learned to attend to ward temperature, power status, water availability, patient crowding, and equipment reliability as practical signals that could alter the sequence and safety of clinical tasks. Across accounts, participants emphasised observing and assisting under supervision, staying firmly within their scope of practice while learning how teams keep patients safe when systems are strained.

On surgical and medical wards, UNS consistently worked with nursing preceptors to adapt routines when heat and outages coincided, noting that air-conditioning often became ineffective once temperatures exceeded 45 °C: *“On hot days we were instructed to bring wound care forward to the morning and keep afternoon tasks light; if the ward temperature climbed, we notified the preceptor and reassessed observations more frequently”* (UNS 04). UMS, typically working under a Senior Resident Medical Officer (SRMO), described role-bounded adjustments that prioritised those most vulnerable to heat stress:

“Under the SRMO’s supervision, we prioritised initial vitals and hydration checks for older patients during heat alerts and escalated any red flags immediately. Also, on days above 40–45 °C, triage is different from the first minute—it changes who you bring in, and how fast you discharge to avoid overcrowding of the emergency department” (UMS 05, Y5).

“When there is a power outage, nurses switch from the electric intravenous pump (IV) to the gravity line so that the generator sustains […]. We have had to change our practice after the generator failed due to overload. This way, only the most vital machines run without interruption.” (UNS 09, Y4)

“For us, it was not always possible to say, ‘this is because of climate change’ or ‘this is only because of the hospital system.’ On the ward, the problem came as heat, then power cuts, then too many patients waiting. Our responsibility as students was to notice the risk, tell the nurse in charge or SRMO, and follow the safer plan” (UMS 03, Y5).

Students framed the 2023 earthquake as a prompt for readiness drills layered onto heatwave routines rather than a separate topic. They learnt evacuation protocol, including who to call and how to keep patients and families safe: *“After the 2023 earthquake, we walked the evacuation route at the start of each rotation; we now know who collected charts, who called maintenance, who stayed with high-risk patients, and who guided families to the assembly point”* (UMS 20, Y5). Similarly, grid fluctuations and equipment alarms were described as infrastructure-related safety concerns that required clear escalation rather than independent student decision-making. On the other hand, routine cold-chain awareness became a shared habit: *“We were told to confirm refrigerator status during grid fluctuations and to alert the nurse in charge if the alarm sounded; students did the checks, the staff made the decisions”* (UNS 06, Y4).

“During a heatwave with intermittent power outages, our SRMO briefed us to keep reviews brief, recheck electrolytes for anyone symptomatic, and write clear paper orders in case the system failed. We followed the plan, kept families informed about delays, and escalated early to the registrar when concerns arose.”— (UMS 13, Y5)

While these institutional protocols provided a framework for predictable stressors, students felt less prepared when faced with multiple, compounding environmental crises that stretched clinical capacity. Their accounts emphasised practical uncertainty: protocols often existed for single events, but students experienced heat, crowding, outages, infection surges, and air-quality or water-related problems as overlapping pressures.

“It was different after last year’s cycle of disasters. First, during the summer, the smoke from the wildfires meant we were managing a spike in asthma and other respiratory complaints. We had barely recovered from that when the monsoon started, bringing floods and a surge of patients with gastroenteritis from contaminated water. You’re dealing with one public health emergency right after another, and the system has no time to reset. Our protocols are designed for a single crisis, not for this kind of relentless, back-to-back pressure.” — (UMS 21, Y5)

The cumulative strain experienced led to the realisation that even predictable seasonal events could result in significant shortages or highlight deficiencies in staffing, supplies, space, and communication. This ongoing state of crisis meant that even routine annual events, such as increases in respiratory infections, triggered acute shortages. Students observed how resources were managed and how fairness was implemented in practice:

“During a surge, we flagged low stock. The preceptor explained that N95s were prioritised for aerosol-generating procedures in ED and ICU; on our ward, we used surgical masks […]” (UNS 02, Y4).

They also recognised that simple, supervised actions protected those at highest risk:

"During a dengue outbreak, the outpatient queue stretched from the ED into the direct sun. It was mostly families and mothers with small children and older patients […] they had no choice but to wait. We moved the most frail-looking patients to a small shaded area. We felt we had to do something—it was a matter of protecting the most vulnerable ones. But we couldn’t just put up a tent or demand a new system. Our role was to record the waiting times, inform the families, and escalate through the proper channels. We did our small part, and the next day, the hospital administration had tents set up. But you see how easily those people could have been ignored." — (UMS 14, Y5)

Across disciplines, participants portrayed a pragmatic apprenticeship in hazard-aware clinical work: noticing hazards early, executing supervised adjustments, documenting carefully, and escalating through defined channels. As one student summarised*, “One of the ‘Good practices’ for us now includes reading the room—temperature, power, crowding—and telling the right person at the right time” (UMS* *18, Y5).*

### Theme 2: professional futures amid planetary risk: safety, reciprocity, and conditional return

Students characterised their choices regarding specialisation and migration intentions as influenced by factors such as occupational safety, access to reliable training environments, remuneration, family responsibilities, and confidence in the applicability of their acquired skills within Nepal’s health system. Environmental and infrastructural challenges—including heat, power outages, seasonal surges, equipment failures, and maintenance gaps—provided a context for these decisions. However, participants typically articulated their choices in practical terms, focusing on their ability to work safely, learn effectively, support their families, and practice in institutions equipped with functioning systems. In the near term, most sought to practise and train in more resilient systems—to learn in places where safety, supply chains, and maintenance are reliable. Over a longer horizon, intentions diverged by discipline. UMS participants more often described possible return as conditional on institutional readiness and recognised roles, whereas several UNS participants spoke of permanent migration as a route to safer work, economic security, and family stability.

Several UMS participants linked outward mobility to specialist training, exposure to reliable systems, and the hope that skills gained abroad could later be used at home. *“I want to specialise abroad and then return. As a doctor here, you carry status but also an obligation. In our hospitals, a lack of planetary awareness is compounded by resource constraints. I feel I should come back when I can bring that experience and expertise with me” (UMS* *19, Y5)*. In these accounts, outward mobility was not described only as a search for prestige; it was also associated with gaining competence in settings where equipment, protocols, supervision, and backup systems were more dependable. However, participants repeatedly stressed that return would be meaningful only if local institutions could support the effective use of those skills.

“Travelling abroad is not solely about securing a more prestigious title. I aim to assess the hospital's operational efficiency, particularly how well the equipment functions, how promptly maintenance requests are addressed, and whether a clear backup plan exists in the event of system failures. However, if I gain these insights yet return to a setting where implementation is not feasible, then what is the use of my learned knowledge?” (UMS 07, Y5).

For many UNS participants, by contrast, the daily realities of resource scarcity, heat alerts, and power cuts were experienced through occupational safety and the right to a predictable, adequately staffed workplace. *“My plan is permanent migration. On the wards during a patient surge, staff safety felt uncertain—PPE was scarce, breaks were shortened, and there was no nurse-to-patient ratio. I want to work in an institution where staffing and supplies are adequate before thinking about coming back to Nepal long term” (UNS 02, Y4)*. Here, migration was described less as an abstract ethical stance than as a practical response to unsafe staffing, insufficient supplies, low remuneration, and limited confidence that workplace protections would improve.

Conditions for return also centred on practical reciprocity: students wanted training abroad to be matched by institutional conditions that would enable them to use acquired skills in Nepal:

“What's the point of learning an advanced surgical technique abroad if I come back and the equipment we need is broken? We see it all the time […]. It becomes a false promise to patients. You can’t say you offer modern care when you can’t even maintain the basics. For me to return, it has to be a genuine partnership. The places that train us must also ensure that we have the right equipment and a robust backup plan when the system fails.”— (UMS 13, Y5).

Participants did not describe reciprocity only as a personal obligation to return; they also framed it as an institutional condition for return to be professionally viable. For some UMS students, this meant robust institutional funding for functioning equipment, maintenance systems, and formal recognition of specialist expertise. For UNS participants, it meant safe staffing, reliable PPE, fair remuneration, and workplaces where nurses were not expected to absorb chronic shortages as normal professional sacrifice.

Several UNS participants grounded their decisions in family responsibility and economic security. Remittances, parental investment, and the prospect of raising safer, healthier families were central to how permanence abroad was justified:

"My parents invested everything in my education. It is my duty to ensure they have a secure and comfortable life as they age. But how can I promise that here, when my salary is so low, and the hospital itself feels so fragile? Then I think about the future […]. Leaving feels like the most responsible choice. It’s not just for me; it’s about providing security for the generation before me and a safer, healthier life for the generation that will come after.” (UNS 08, Y4).

Among UMS students who expressed a willingness to return, specific criteria regarding timing and circumstances were identified. Their return should be aligned with an institution capable of effectively leveraging their specialised skills, ensuring robust operational systems, and demonstrating preparedness for recurrent challenges, including extreme heat, flooding events, public health outbreaks, and potential infrastructure vulnerabilities.

“My dream is to teach a climate-smart medicine, one that's prepared for the realities of Nepal's future—floods, heat, and new diseases [...]. But you can't be a teacher if there's no classroom. The government needs to meet us halfway. It must value this expertise, create roles for us, and show it’s serious about adapting to changing needs. Otherwise, they are asking us to come back and shout into the wind. And that’s not just a bad career move; it’s a betrayal for the next generation of patients!” (UMS 16, Y5).

When viewed collectively, students recognised speciality selection and migration as ethically important paths shaped by planetary risks and the capabilities of institutions. Foundations for the concepts of "return" or "permanence" rested on principles such as workplace safety, equitable access to operational equipment, partnerships that go beyond mere skill acquisition, and a responsibility to both current and future patients.

### Theme 3: navigating curricular gap with student-led solutions

Across accounts, students reported a gap between formal ethics teaching and the practical decisions they encountered during placements under heat, outages, water insecurity, seasonal patient surges, and resource constraints. They did not describe this as an absence of ethics teaching altogether; rather, they described formal teaching as concentrated on classical, case-by-case dilemmas—autonomy, consent, confidentiality—while offering limited preparation for situations in which safety, fairness, infrastructure, and escalation intersected. Yet this silence did not signal indifference. Participants identified concrete, practicable entry points for teaching and assessment, which are presented here as student-generated suggestions rather than as evidence of an evaluated curriculum reform programme.

They described ethics content as decontextualised—imported scenarios with scant relevance to the hazards they actually meet.

"We learn about medical ethics from American and British textbooks. The examples are about patient confidentiality or complex genetic testing. We never once had a class that asked, ‘What is our ethical duty when the monsoon floods the village of the patient we are about to discharge?’ or ‘How do you allocate a handful of antibiotics when a whole community is sick from contaminated water?’ Our problems are different, but our ethics teaching is from another world." — (UMS 18, Y5)

According to students’ accounts, this disconnect left them underprepared for decisions in which fairness, risk, and infrastructure converged during routine clinical work**.** A nursing student put it plainly:

“We don’t have a module that connects heat, power, water, and fairness. It was mostly isolated talks. On the ward, you meet all of them at once, and you need to know whom to tell, what threshold triggers action, and how to explain delays to families” (UNS 06, Y4).

UMS participants suggested case-based teaching that would specify thresholds, role responsibilities, and escalation steps when resources become limited and risks increase. Rather than asking students to solve institutional problems independently, these cases would help clarify what students should notice, document, explain, and escalate within their scope of practice.

“A power failure protocol must prioritise the protection of the NICU (Neonatal Intensive Care Unit) and maintain the integrity of the cold chain. Following this, it is essential to provide a rationale for deferring non-urgent imaging procedures. This approach not only underscores patient safety but also ensures equitable treatment across the patient population.” (UMS 19, Y5).

Some participants also wanted teaching to move beyond retrospective crisis response and include anticipatory systems thinking: how environmental exposures, infrastructure limits, and clinical case-mix are connected, and how trainees can act within their role before risk escalates. One UMS participant connected this educational need to the desire to learn a more preventive way of thinking:

“Returning is a duty for me. As a doctor in Nepal, you have a responsibility that goes beyond the clinic; you serve the community. But right now, I see our hospitals are stuck in a cycle of just reacting to crises. We don't connect the dots between the rising heat and the increase in kidney disease, or between the polluted water and the endless gut infections. I feel I need to go abroad to learn a different way of thinking—how to build a system that anticipates these problems. My obligation isn't just to return with better clinical skills, but to bring back a way to help us prevent these environmental health disasters before they happen.” (UMS 14, Y5)

Students highlighted the critical role of assessment in shaping learning outcomes. They advocated for OSCE stations designed to simulate realistic system failures. In these scenarios, candidates would be required to explain safe adjustments, identify the limits of their student role, document the rationale for decisions, and select an appropriate escalation pathway.

“If an OSCE station examines system failures—such as trauma scenarios, heat, outages, or the shortages of PPE—and requires us to justify safe adjustments and select the appropriate escalation channel, we will be prepared for it when it occurs in real life.” (UMS 14, Y5).

They also proposed inter-professional drills—nursing, medicine, biomedical engineering —so roles and hand-offs are practised, not presumed:

“Seeing how SRMO, the medical superintendent in charge, and the maintenance department coordinate during an outage would teach us where our role begins and ends” (UMS 19, Y5).

Finally, students argued that ethical awareness must extend beyond the bedside to the immediate care environment—including air quality, waste management, water reliability, heat exposure, equipment functionality, and cleaning systems. They linked this learning need to the practical knowledge of staff whose expertise is often absent from formal teaching but is central to patient safety.

“The most eye-opening session we had was when they invited cleaners, ward assistants, maintenance team members, biomedical engineers, and hospital scientists to speak with us. We discuss infection control in theory, but they understand the reality—they know the airflow mechanisms from one end of the ward to the other, or the exact path contaminated waste takes. Listening to them made these invisible environmental risks feel real for the first time. It was a powerful lesson: to truly understand patient safety, you have to respect and listen to the expertise of the people who are on the frontline of those risks every single day.” (UNS 07, Y4).

These findings are reported as student-perceived curricular gaps and student-generated solutions. Their broader implications for planetary health ethics education, curriculum implementation, and institutional preparedness remain pressing frontiers that require ongoing translation into practice and institutional validation.

## Discussion

The interpretation that follows grounds the students’ practice-proximal accounts within the planetary health ethics framework [15], reinforced by the World Health Organisation’s competency framework for health professionals [51]. Rather than treating environmental disruption as background context, this analytical lens systematically maps the framework’s core commitments onto the study’s empirical findings.

First, students’ descriptions of supervised safety work under infrastructural and environmental stress are analysed as vital acts of *hazard recognition*, explicitly drawing heat, power outages, water reliability, vector dynamics, and seismic risk into the clinical picture. Second, their narratives regarding professional futures—which weigh occupational safety, institutional readiness, reliable training, and family responsibility—are read as active attempts to navigate *precaution and fair allocation* under systemic constraint, balancing *immediate and intergenerational obligations* alongside the *environmental externalities* of care. Third, the students’ proactive identification of curricular gaps and their proposed solutions—such as hazard-specific OSCE stations, drills, and escalation pathways—demonstrate an emergent understanding of *advocacy within governance* as a legitimate professional duty. Finally, their specific suggestions for locally grounded cases directly fulfil the framework's mandate for epistemic inclusion, elevating *situated expertise* and marginalised voices in defining safe and just practice. While this interpretive framework is expansive, the empirical scope of this analysis remains strictly clinical and educational; broader claims regarding national governance, regulation, or operational sustainability are treated carefully as bounded implications rather than direct findings.

Building from this bounded interpretation, the findings suggest that clinical ethics in this setting must attend to multi-factorial instability rather than climate exposure alone. Students described hazard signals—heat, power and water reliability, dengue-season surges, cold-chain alarms, and earthquake-readiness routines—as practical features of ward work shaped by overlapping environmental, infrastructural, seasonal, seismic, and health-system pressures. Climate change is best understood here as an intensifying force for heat, hydrometeorological stress, air quality, and vector-borne disease, not as the cause of earthquakes, power interruptions, governance weakness, supply-chain fragility, or equipment failures [5, 52]. Treating these conditions as mere background obscures the supervised safety work trainees described: anticipating risk, stewarding scarce resources, documenting decisions, and escalating concerns through defined channels. Planetary health ethics helps interpret why such infrastructures matter ethically: they shape who is protected and who remains exposed [7].

The next issue is how these infrastructures shape fairness in practice. Students reported deferring non-urgent procedures during outages, protecting the cold chain, allocating PPE during seasonal surges, moving vulnerable patients out of prolonged heat, documenting waiting times, and escalating concerns through defined channels. These actions should not be read automatically as planetary health ethics; they were first pragmatic, supervised efforts to keep patients safe. They become ethically salient when they require transparent criteria for prioritisation, precaution under uncertainty, accountability for trade-offs, and protection of those least able to avoid exposure. Ethics teaching and professional codes should therefore make explicit who is prioritised, at what trigger point, and why, while normalising documentation that renders constrained decisions visible for scrutiny and learning. This aligns planetary health ethics with health equity and environmental justice by recognising that the acute burdens of infrastructural and ecological disruption inevitably fall on the most marginalised populations [53, 54].

This concern with fairness extends to professional futures, yet the data indicate that such mobility is anchored more in pragmatic and structural realities than in explicit planetary-ethical reasoning. Students described their choice of speciality and migration decisions in terms of occupational safety, remuneration, family responsibilities, reliable training, usable equipment, maintenance systems, and conditions for return. These accounts align with health labour market perspectives, in which professionals seek job security, development, and safer practice environments [55, 56], and with evidence that technology-intensive care reshapes labour markets and skill expectations [57]. Intergenerational responsibility remains relevant, but it was grounded in family obligations, parental investment, remittances, and secure futures rather than abstract planetary reasoning. Thus, “brain drain” is better discussed in terms of reciprocity and structural inequality [58]: skills can benefit Nepal only when equipment, protected teaching time, maintenance, and recognised roles make a return viable. When this reciprocity fails, moral injury may emerge [59], underscoring the need to align international education and domestic planning [17, 60].

That alignment also depends on whose knowledge counts in education. Students reported that cleaners, ward assistants, maintenance staff, biomedical engineers, and hospital scientists made visible airflow, waste pathways, cleaning systems, equipment vulnerabilities, and environmental risks that formal lectures often missed. Their accounts therefore support a bounded curricular implication: include situated expertise, as it helps trainees understand patient safety in light of environmental and infrastructural constraints. Interpreted cautiously, this is an instance of epistemic justice—treating often-overlooked staff not as symbolic contributors, but as warranted knowledge-holders whose expertise improves safety and legitimacy [16, 61].

This attention to situated expertise also redirected students’ ethical attention to the immediate care environment. Rather than demonstrating multispecies justice in full, their accounts showed how air quality, waste management, water reliability, cleaning systems, heat exposure, vector-borne risk, and equipment performance shape patient safety. These observations point to environmental externalities of care: measures that protect individual patients may still shift harm to neighbouring communities or non-human life through smoke exposure, contaminated run-off, or poorly managed waste [62–64]. Health care’s environmental footprint is well documented [65], but this dataset supports a narrower claim: ethics education should help trainees recognise environmental risks visible in clinical work and know when to escalate them. Broader multispecies and ecological implications remain important, but require further empirical study [66, 67].

Accordingly, governance and regulatory alignment should be conceptualised as secondary implications rather than primary empirical outcomes. Students’ emphasis on clear escalation pathways, drills, and role-bounded responsibilities supports immediate education-level and institution-facing actions: specifying hazard thresholds, decision flows, documentation expectations, and escalation routes for scenarios such as power failure affecting the NICU or cold chain. Embedding such elements in curricula, OSCEs, interprofessional drills, and supervisory protocols is consistent with calls to integrate planetary health into health-professional education [68–70]. However, broader claims about accreditation, licensing, hospital policy, and professional codes require additional evidence from curriculum audits, educator interviews, implementation studies, and policy analysis. Establishing this analytical boundary preserves the empirical integrity of the students' accounts while maintaining their actionable relevance for institutional design.

Within these delineated clinical parameters, communication and advocacy are best understood as role-bounded clinical practices rather than ancillary moral aspirations. Participants described brief factual notes during heat alerts, documentation of waiting times, escalation through preceptors, SRMOs, and registrars, and measured explanations to families about delays. These practices protected patients while respecting confidentiality, hierarchy, and student scope. Planetary health ethics supports this procedural view of advocacy, in which trainees learn when and how to raise environmental or infrastructural risks without assuming authority they do not hold [60, 68]. Training in health promotion, science communication, and climate-health advocacy equips practitioners to clearly explain the rationale behind difficult trade-offs, ensuring that resource-constrained decisions are understood as necessary precautions rather than arbitrary actions [71–73]. Consequently, the imperative to confront systemic environmental hazards is more accurately conceptualised as a competency for advanced professional practice, rather than an immediate ethical burden placed upon trainees.

This analytical scope extends to the domain of operational ethics. While students did not explicitly evaluate institutional carbon accounting, procurement policies, or macro-environmental performance frameworks, their narratives underscore that trainees absorb the ethical priorities modelled by their institutions. Essential infrastructural elements—such as backup power generation, cold-chain reliability, safe waste management, and timely maintenance—function as a potent *hidden curriculum*, as they ultimately dictate the feasibility of safe care. Consequently, the imperative to reduce healthcare’s environmental footprint must be conceptualised as a vital trajectory for future institutional research and policy development that extends beyond the immediate clinical purview captured in these accounts [74, 75].

Taken together, these findings reposition high-hazard LMIC settings not merely as passive testing grounds for planetary health ethics, but as vital sites of theoretical and practical innovation. The students' accounts reveal that clinical pedagogy, professional trajectories, and curricular expectations are fundamentally shaped by a baseline of multifactorial instability—a precarity that climate change multiplies rather than originates. For immediate educational practice, this necessitates the structural integration of locally grounded case studies, defined hazard thresholds, specialised OSCE stations, interprofessional drills, formalised escalation pathways, and the inclusion of situated expertise. While broader institutional and regulatory reforms remain critical, they must undergo rigorous empirical validation before being advanced as actionable policy mandates.

### Limitations and future research

This study presents several limitations that affect the interpretation of the findings and highlight areas for future research. It draws on a single semi-urban tertiary institution in southern Nepal and a final-year student sample, which limits transferability to earlier stages of training, other regions of Nepal, and rural or urban facilities whose hazards, staffing models, infrastructure, and resources may differ materially from those of semi-urban hospitals. The gender and discipline composition reflects the programme and workforce context but constrains certain cross-group contrasts. Findings rely on self-reports within active training relationships, so social desirability and power dynamics may have muted dissent or inflated confidence in escalation. Because participants were trainees who primarily observed or assisted under supervision, their accounts cannot substitute for the decision-making perspectives of registered nurses, medical officers, educators, preceptors, hospital managers, facilities teams, or policy actors.

The study is also limited by its empirical scope. It explored final-year students’ experiences, meanings, and curriculum-relevant suggestions; it did not directly examine formal curricula, educator practices, assessment blueprints, accreditation processes, hospital governance, or operational sustainability systems. Accordingly, the findings are best understood as student-informed, practice-proximal insights and as a basis for curriculum-relevant hypotheses, not as evidence that any programme-level intervention has been implemented or evaluated. The study was cross-sectional and conducted during a defined period, whereas heat, vector-borne disease, flooding, power interruptions, water insecurity, and seismic preparedness vary across seasons and years. The analysis also treated climate change as one intensifying pressure within broader multi-factorial environmental, infrastructural, social, and health-system instability; the design cannot attribute specific clinical disruptions to climate change alone. Perspectives from patients, families, communities, environmental services staff, and institutional leaders were not included, limiting triangulation.

Future research should therefore be multi-site and mixed-methods, including public and private institutions across Terai, hill, and mountain settings. Longitudinal cohorts should follow students from the final year through internship and early practice to examine how preparedness, escalation behaviours, safety incidents, and migration or return intentions evolve. Ethnographic observation, service-level data, and document analysis could clarify how hazard signals are recognised and acted upon in real time. Curriculum audits, educator and preceptor interviews, assessment blueprint reviews, and governance analyses are needed to test whether the gaps identified by students are reflected in formal educational and institutional structures. Intervention studies should pilot and evaluate locally grounded cases, OSCE stations simulating system failures, interprofessional drills, threshold-based escalation tools, and role-bounded advocacy teaching, with outcomes including knowledge, confidence, documentation quality, appropriate escalation, teamwork, and feasibility. Future studies should also assess the implementation conditions for these recommendations, including faculty capacity, institutional resources, maintenance systems, and policy levers such as accreditation and licensing. Co-design with students, registered clinicians, ward assistants, maintenance staff, biomedical engineers, hospital scientists, and community representatives would strengthen epistemic inclusion while ensuring that proposed reforms remain clinically usable and institutionally realistic.

## Conclusion

Health professional education in high-hazard settings in LMICs must equip graduates to navigate systems in which clinical judgment is intricately linked to infrastructure, environmental conditions, and institutional preparedness. The core message of this study is not that climate change alone creates these challenges, but rather that it exacerbates longstanding and multifaceted instability that already influences clinical education, professional trajectories, and patient safety. For future nurses and doctors, ethical practice goes beyond recognising dilemmas at the bedside; it requires knowing how to respond safely and fairly, within their scope of practice, when factors such as heat, power outages, equipment failures, or evacuation risks affect the care that can be delivered.

The practical takeaway is clear: planetary health ethics becomes meaningful when translated into teachable and assessable routines. Curricula should help students recognise hazard thresholds, document constrained decisions, communicate transparently, escalate appropriately, and learn from the staff who understand the material conditions of care. Institutions, in turn, must model the preparedness they expect graduates to practise. In this sense, high-hazard settings are not peripheral to planetary health ethics; they are vital sources of insight for educating health professionals capable of delivering just, resilient, and context-responsive care.

## Data Availability

The data supporting this study's findings are available on request from the corresponding author. However, the data is not publicly available due to privacy or ethical restrictions.
